# Fellow-Workers and the Roll of Honour

**Published:** 1915-09-11

**Authors:** 


### ROUND THE WORLD.

[The Editor invites Early Information and Contributions Marked "Fellow-Workers."]

### Fellow-Workers and the Roll of Honour.

Lieutenant-Colonel George Alfred Edsell, who has lately died at home as the result of an illness contracted whilst on service abroad, had been in practice at Surbiton for some years. Always interested in military work, he had reached a responsible position in the Territorial branch of the R.A.M.C., and been placed in command of the 1/3rd Home Counties Field Ambulance, with which he went abroad several months ago. Studying medicine at University College and St. Bartholomew's Hospital, Dr. Edsell qualified in 1886, and later took the M.D. Durh., as well as the D.P.H. of both Cambridge and the R.C.S. Eng. After qualification he held the posts of resident obstetric assistant and clinical assistant in the throat, eye, and skin departments at "Bart.'s," whilst on settling in practice he became honorary surgeon to the Home for Cripples at Surbiton.

Second-Lieutenant James Gilbert Sutherland, who has died of wounds, was working through the medical curriculum at Edinburgh when the war broke out. His intention was to become a dental surgeon, in which capacity he believed his hand would be strengthened if he also obtained medical and surgical qualifications. Already a corporal in the Edinburgh O.T.C., he obtained a commission in August 1914, and after training went to the Front in the early weeks of the present summer. Mr. Sutherland, who had been attached to the Highland Light Infantry, had taken the M.A. degree of Edinburgh University.

The New Zealand Army Medical Corps has lost a distinguished officer by the death of Major Thomas Copeland Savage, who is reported to have died in Egypt at the New Zealand Military Hospital, Pont Koubba. A student of University College Hospital and Medical School, he had done very well in early years, taking a gold medal and scholarship in anatomy (1898), and the Atkinson Morley scholarship in surgery (1901). This promise of future distinction he followed up by winning the gold medal at the B.S. Lond. in 1901, the year after he qualified and became an M.B. Lond. Almost immediately after Major Savage took the F.R.C.S. Eng. and devoted himself to practical surgery and ophthalmology. His next step was to settle in Auckland, New Zealand, where he became honorary surgeon to the General Hospital, consulting surgeon to the North Auckland Hospital, and examiner in surgery for the University of New Zealand. Last year he volunteered for military service, and went to Egypt with the New Zealanders.

Captain Arthur Kellas, R.A.M.C. (T.), whose name has also appeared in a Dardanelles casualty list, was an Aberdeen student who qualified by taking the M.B., Ch.B. there in 1906. Subsequently Captain Kellas, who was a son of the late Mr. J. F. Kellas, superintendent of mercantile marine at Aberdeen, took the D.P.H. diploma of his University, and held the posts of resident physician and surgeon at the Royal Hospital for Sick Children, Aberdeen. Later he joined the staff of the Aberdeen Royal Asylum, where he was senior assistant physician when the call came for more Army surgeons.

Captain James Noble Armstrong, R.A.M.C., who recently fell in action on the Continent, qualified in Dublin last year by taking the M.B., B.Ch. degrees. During his student career at Trinity College, Dublin, he won a senior moderatorship and gold medal in natural science. Joining the Army, he served for a time at the base hospitals, and then became attached to the Durham Light Infantry.

The sad death of the late Mr. Quarry Silcock, F.R.C.S., of St. Mary's, just as he had attained a position of distinction amongst London surgeons, is recalled by the loss of his younger son, Second-Lieutenant Bertram Baber Silcock, of the Royal Welsh Fusiliers, killed in action at the Dardanelles. With the intention of following his father's profession, the late Mr. Bertram Silcock entered University College Hospital Medical School, but had not qualified when war broke out. However, he had had previous experience of military surgery as a British Red Cross dresser with the Greeks during the late Balkan wars, his services then being recognised by the award of the Cross of the Knight of the Order of Our Saviour by the King of the Hellenes. On the outbreak of the Great War he first went as surgeon-probationer on the hospital ship *Rohilla*, but subsequently joined the combatant forces and, obtaining a commission this summer, was sent to the Front about a month ago.

The British ambulance which is going to the assistance of the wounded on the Italian Front will have two distinguished British practitioners, Dr. W. R. Dakin and Dr. G. S. Brock, as surgeon and physician respectively. This unit, which is to be under the direction of Mr. George Trevelyan, the well-known authority on Garibaldi, has sixteen ambulances given by the British Red Cross Society and four provided by the Society of Friends.

Dr. William Radford Dakin, who retired from active work some little time since, was for many years known as one of our leading gynæcologists and obstetricians. A student of Owens College, Manchester, and later of Guy's Hospital, he qualified in 1882, subsequently becoming an M.D., B.S. Lond., and F.R.C.P. Lond. Dr. Dakin's brilliant work carried him on to the staff of St. George's Hospital, with which he maintained a distinguished connection extending over a long period, and in the course of which he wrote the popular text-book of midwifery which has been so useful a companion to thousands of students.

Dr. G. Sandison Brock, who will be Dr. Dakin's colleague with the British ambulance in Italy, is a prominent member of the English community in Rome, where he is physician to the British Embassy. An F.R.C.S.E., he graduated at Edinburgh in 1880, and won a gold medal at the M.D. Edin. in 1892. Subsequently Dr. Brock took the M.D. of the University of Rome.

Lieutenant H. Chell, who died of wounds in Flanders, and Second-Lieutenant P. S. Snell, killed in the recent Dardanelles fighting, were both medical students who interrupted their academic work to fight for their country. The former was working at St. Mary's Hospital, Paddington, and, receiving a commission, eventually became attached to the Royal Fusiliers, whilst Mr. Snell was a Dublin student well known as an athlete and first-class cricketer.

